# Air Germs at Different Seasons

**Published:** 1883-04

**Authors:** M. Louis Olivier


Air Germs at Different Seasons.—The observations conducted at the Observatory of Montsouris show that there are on the average eighty bacteria in a cubic metre of air. The highest number was observed in the fall, the lowest in the winter. There were found fifty bacteria in December and January, only thirty-three in February, one hundred and five in May, fifty in June, and one hundred and seventy in October. The diagrams of daily observations show that the number of spores of these algæ increases with the temperature. Inversely to what takes place in the case of the molds, the number of the schizophytes, small in rainy weather, rises when all the moisture has disappeared from the surface of the soil. The counteraction of moisture is stronger than the direct action of temperature ; and this fact accounts for the rarity of the bacteria after the great rains of February, April and June. Still a long period of dry weather does not appear to be favorable to the development of the plants. The number rises at first during the hot season, but diminishes under the influence of a progressive desiccation toward the second or third week.

The diminution in hygrometric conditions manifested in September and October explains the recrudescence of the bacteria during these months. Some micrographers have suggested that the germs may be transported by the vapor of water ; but M. Miquel's experiments invalidate this hypothesis, and indicate that the evaporation of water from the surface of the ground never carries any schizophytes with it. On the other hand, numerous tests have shown that dry dusts, especially those of hospitals, proceeding from substances in a state of putrefaction, sanious pus, and the dejections of the sick, are charged with microbes. Great agglomerations of men furnish the most of them. According to the measurements made in the Rue de Rivoli and Montsouris, the air in the interior of Paris is nine or ten times richer in bacteria than that in the neighborhood of the fortifications.—M. Louis Olivier, *in Popular Science Monthly for May.*

